# Relationship Between COVID-19 Information Sources and Attitudes in Battling the Pandemic Among the Malaysian Public: Cross-Sectional Survey Study

**DOI:** 10.2196/23922

**Published:** 2020-11-12

**Authors:** Emma Mohamad, Jen Sern Tham, Suffian Hadi Ayub, Mohammad Rezal Hamzah, Hasrul Hashim, Arina Anis Azlan

**Affiliations:** 1 Centre for Research in Media and Communication Faculty of Social Sciences and Humanities Universiti Kebangsaan Malaysia Bangi, Selangor Malaysia; 2 Department of Communication Faculty of Modern Languages and Communication Universiti Putra Malaysia Seri Kembangan, Selangor Malaysia; 3 Department of Communication School of Arts Sunway University Petaling Jaya, Selangor Malaysia; 4 Centre of Excellence for Social Innovations and Sustainability Faculty of Applied and Human Sciences Universiti Malaysia Perlis Kangar, Perlis Malaysia

**Keywords:** COVID-19, information source, confidence, media, social media, government, Malaysia, online information, survey

## Abstract

**Background:**

There are multiple media platforms and various resources available for information on COVID-19. Identifying people’s preferences is key to building public confidence and planning for successful national health intervention strategies.

**Objective:**

This study examines the sources of information for COVID-19 used by the Malaysian public and identifies those that are associated with building public confidence and positive perceptions toward the Malaysian government.

**Methods:**

A cross-sectional online survey of 4850 Malaysian residents was conducted. Participant demographics, media use, information sources, and attitudes surrounding COVID-19 were assessed. Descriptive statistics and multiple logistic regression analyses were conducted to gauge the relationship between demographics, information sources, and attitudes toward COVID-19.

**Results:**

Malaysians primarily used television and internet news portals to access information on COVID-19. The Malaysian Ministry of Health was the most preferred source of COVID-19 information. Respondents who referred to the Ministry of Health, television, and the Malaysian National Security Council for information were more likely to believe that the country could win the battle against COVID-19 and that the government was handling the health crisis well compared to those who referred to other information sources. Those who used the World Health Organization, friends, YouTube, family, and radio as sources of information were less likely to harbor confidence and positive belief toward combating COVID-19.

**Conclusions:**

Managing information and sustaining public confidence is important during a pandemic. Health authorities should pay considerable attention to the use of appropriate media channels and sources to allow for more effective dissemination of critical information to the public.

## Introduction

The COVID-19 health crisis has introduced new challenges to effective health information delivery around the world. Past research has indicated that media hype surrounding a health crisis has caused panic and uncertainty among the public [1]. In the context of COVID-19, this is exacerbated by the misinformation and disinformation surfacing on social media. Previous studies have shown that self-efficacy plays an important role in predicting the health behaviors of the public [2]. However, in a pandemic situation, there is an increased reliance on the government and health authorities to manage the problem. The need to provide clear, consistent, and credible information surrounding the COVID-19 pandemic is key to disease mitigation and control.

One study has revealed that health information behaviors in Malaysia are highly situational and that research on health information seeking in Malaysia is still very limited [3]. Health information seeking behaviors and sources differ based on the type of disease and demographic group. Local studies on the H1N1 pandemic have found that Malaysians gathered information from the news media, social media, and authorized websites, such as the MyHealth Portal under the Ministry of Health (MOH) [2,4]. A separate study found that a majority of respondents felt that the internet was useful for assisting them in making health decisions [5].

It has been almost 1 year since COVID-19 emerged at the end of 2019. Countries all over the world are at various stages of disease mitigation. As of October 29, 2020, Malaysia has recorded 29,441 total COVID-19 cases, with 18,499 recoveries and 246 total deaths [6]. In terms of disease management, Malaysia is perceived as one of the successful countries, and this is largely due to the implementation of the Movement Control Order (MCO) on March 18, 2020 [7-9]. The country has since progressed to the third stage of the MCO called the Recovery MCO, which began on June 10, 2020. Under the Recovery MCO, Malaysians have been allowed to return to work, schools and businesses have been allowed to reopen, and travel restrictions have been loosened.

Since the implementation of the MCO, the Malaysian public has been dependent on both local and international media for information related to COVID-19. Official information and local updates on the disease were broadcast via press conferences and regular messaging through multiple telecommunication channels, including traditional media, digital media platforms, and social media. In Malaysia, these channels include government and privately owned media.

Although information is available through various local and international sources, the MOH Malaysia and the Malaysian National Security Council (NSC) are the official sources responsible for communicating information about COVID-19 and its control in the country. As such, both the MOH and NSC have made concerted efforts to maintain a consistent and reliable flow of information to the public through both traditional and new media sources. Credible spokespeople from the MOH and NSC are chosen to deliver COVID-19 updates to ensure the clarity, trustworthiness, and congruence of information. Never before has Malaysia faced a health crisis that has required a response of this scale. Current literature emphasizes the importance of communication in a crisis for public reassurance and order [10,11]. The significant role of local media in communicating messages to the public during the MCO has also been highlighted, specifically in urging people to stay home, practice good personal hygiene, and observe social distancing when in public areas [12].

In Malaysia, the challenge of the pandemic was further complicated by the resignation of then-Prime Minister Mahathir Mohamad on February 24, 2020. The sudden change of government that occurred as the threat of COVID-19 grew introduced new uncertainties to an already volatile situation. The new government faced heightened pressure to instill confidence and trust among the people [13]. The government’s ability to manage the spread of the virus and its capacity to provide quality health care for patients were important components for building that trust.

With the enforcement of the MCO, the Malaysian authorities demonstrated that they viewed the situation seriously and intended to take the necessary action to curb the spread of the virus [14]. However, implementing strict MCO rulings carries its own risks. Citizens in countries around the world have responded variably to lockdown and control measures [15]. Some have acted in retaliation to these measures, citing distrust and skepticism in health authorities [16].

Strict enforcement of the MCO by Malaysian authorities, such as the NSC and the Royal Malaysia Police, could have potentially caused backlash from the Malaysian public. However, its implementation has resulted in improved compliance with the MCO restrictions [17]. Additionally, a recent study has indicated that the Malaysian public has a positive perception on the ability of the government to control the spread of COVID-19 and high confidence that the country could win the battle against the pandemic [18].

However, this confidence and positive perception are not without challenges. This study examines where the Malaysian public source their information on COVID-19 and who they refer to for reliable information. Subsequently, this study identifies sources that are more likely to contribute to higher confidence in combating the pandemic and positive perceptions toward the government among the Malaysian public.

## Methods

### Participants

This cross-sectional study was conducted online between March 27 and April 3, 2020. At the time of data collection, the number of local COVID-19 cases increased from 2161 on March 27, 2020 to 3333 on April 3, 2020 (out of a 32 million total population). The survey was conducted 1 week after the highest R-naught (R0=3.55) was recorded, which means that 1 infected person on average will transmit the disease to 3.55 people [19].

During the survey period, Malaysia was under the MCO. Hence, it was not feasible to conduct a systematic nationwide sampling procedure. In order to overcome this challenge, the researchers distributed an online survey using the Survey Monkey website. Survey participants were recruited by posting a 1-page recruitment message onto authors’ Facebook, Twitter, Instagram, and WhatsApp groups with community leaders and social media influencers. The recruitment message contained a brief introduction on the purpose of the study, the procedures of data collection, the voluntary nature of participation, declarations of anonymity and confidentiality, and notes for filling in the questionnaire, as well as links for English and Malay language versions of the online questionnaires. Members of the public aged ≥18 years and those who resided in Malaysia were eligible to participate in this study. The target sample size was 3640, which was determined by identifying the smallest acceptable size of a demographic subgroup with a 5% margin of error and 95% confidence level. A total of 5137 surveys were initiated. Only surveys that were missing less than 10% of data were retained [20]. A total of 4850 responses were included in the final analysis.

This study was approved by the Institutional Review Board of Universiti Kebangsaan Malaysia (JEP-2020-276). The study participants were given no incentive for participation. Participants gave consent to willingly participate in the survey by clicking the “continue” button, which would direct them to complete the self-administered questionnaire.

### Measures

The questionnaire was developed based on previous studies [21]. The first part of the survey questionnaire involved collecting demographic data on respondents’ sex, age, ethnicity, current residing state, locality (urban or rural), occupation, and household income. The second section recorded the kinds of media and sources participants used to obtain information about COVID-19. With regard to media, participants could select any of the following: television, radio, online news portals, WhatsApp, Facebook, Twitter, Instagram, and YouTube. With regard to main sources, participants could choose the MOH, WHO, NSC, family, or friends. The choices for media and main sources were thereafter categorized into the following: mass media, social media, interpersonal sources, and authorities. Answers were coded as “yes”=1 and “no”=0. The third section measured how confident respondents were in battling the COVID-19 pandemic based on the following 2 questions: (1) “Do you have confidence that Malaysia can win the battle against COVID-19?” and (2) “The government of Malaysia is handling the COVID-19 health crisis very well.” Responses to these questions were coded as “no”, “disagree”, and “I’m not sure” responses equaling 0 and “yes” and “agree” responses equaling 1. The instrument can be found in Multimedia Appendix 1.

### Data Analysis

The collected data were analyzed using SPSS software version 26 (SPSS Inc). The proportion of participants who used each common source to obtain information about COVID-19 was presented in terms of number and percentage. To evaluate whether demographic variables (ie, sex, age, occupation, current residing state, locality, and household income) were associated with the source of information used for COVID-19, we performed a series of logistic regression tests using the forward logistic regression method. For this method, each variable is entered into the model and its value assessed based on the likelihood ratio estimates. If the variable makes a significant contribution to the model, then it is retained, and vice versa [22,23]. Logistic regression was also conducted to examine the relationships between the source of information used for COVID-19 and respondents’ confidence and positive attitude toward the government in handling the health crisis. ORs, 95% CIs, and their corresponding *P* values are reported as indicators of the magnitude and statistical significance of associations. A *P* value of <.05 was considered statistically significant for all analyses.

## Results

In total, the data of 4850 respondents (female: n=2808, 57.9%; male: n=2042, 42.1%) were analyzed. Most of the sample were literate and able to complete the questionnaire. Table 1 presents the descriptive statistics for respondents’ demographic characteristics and the sources they used for obtaining COVID-19 information. The median age of respondents was 33.00 years (range 18-73 years). Most respondents (4026/4850, 83%) were Malay/Bumiputera, and 70% (2295/4850) of respondents resided in urban areas.

The Malaysian MOH was the most referred to source of information for the majority of study respondents (1540/4850, 95.1%) during MCO implementation in Malaysia, followed by television (3542/4850, 73%) and online news portals (3125/4850, 64%). A very small proportion of the participants obtained their information from interpersonal sources, such as friends (1235/4850, 26%) and family (1065/4850, 22%).

Tables 2-4 present the logistic regression results on the associations between demographic characteristics and each source of information. The results indicated that Malay/Bumiputera and Indian women had more than 1.7 times the odds of using television (OR 2.4, 95% CI 1.5-3.8; OR 1.7, 95% CI 1.0-2.9, respectively) as an information source compared to women of other ethnicities. Compared to men, women had 1.2 times the odds of using online news portals to seek COVID-19–related information (OR 1.2, 95% CI 1.1-1.4). In terms of social media use, the results were mixed. Women aged 30-49 years were 1.6 times more likely to use Instagram and 1.7 times more likely to use Telegram as sources of information for COVID-19 than men aged >50 years (OR 1.6, 95% CI 1.2-2.1; OR 1.7, 95% CI 1.1-2.6, respectively). The results also showed that family and friends were not the preferred source of information for COVID-19.

**Table 1:** Descriptive statistics for participant demographic characteristics and the sources participants used for obtaining COVID-19 information (N=4850).

Variables		Statistics
**Sex, n (%)**		
	Male	2042 (42.1)
	Female	2808 (57.9)
Age (years), median		33.00
**Ethnicity, n (%)**		
	Malay/Bumiputera	4026 (83)
	Chinese	511 (10.5)
	Indian	231 (4.8)
	Other^a^	82 (1.7)
**Locality, n (%)**		
	Urban	3395 (70)
	Rural	1455 (30)
**Occupation, n (%)**		
	Public servant	2173 (44.8)
	Student	1125 (23.2)
	Private sector	955 (19.7)
	Self-employed	267 (5.5)
	Not employed	195 (4)
	Retiree	96 (2)
	Other^b^	32 (0.7)
**Income^c^** **(RM/month), n (%)**		
	≤3000	1540 (31.8)
	3001-6000	1289 (26.6)
	6001-9000	832 (17.2)
	9001-12,000	575 (11.9)
	≥12,001	614 (12.7)
**Sources of information about COVID-19, n (%)**		
	Ministry of Health	4614 (95.1)
	Television	3542 (73)
	Online news portal	3125 (64.4)
	Malaysian National Security Council	3069 (63.3)
	Facebook	2993 (61.7)
	WhatsApp	2837 (58.5)
	World Health Organization	2295 (47.3)
	Twitter	1281 (26.4)
	Instagram	1278 (26.4)
	Friends	1235 (25.5)
	YouTube	1080 (22.3)
	Family	1065 (22)
	Radio	854 (17.6)
	Telegram	344 (7.1)

^a^Other ethnicities included Punjabi, Bugis, and Eurasian, just to name a few.

^b^Other occupations included manual labor and contract/part-time work.

^c^A currency exchange rate of RM1=US $0.24 is applicable.

**Table 2:** Factors related to the use of COVID-19 mass media information sources.

Variables		Television		Online news portals		Radio	
		OR (95% CI)	*P* value	OR (95% CI)	*P* value	OR (95% CI)	*P* value
Sex - Female^a^		1.188 (1.037-1.360)	.01	1.223 (1.079-1.386)	.002	0.785 (0.672-0.918)	.002
**Age (years)^b^**							
	18-29	0.423 (0.315-0.568)	<.001	—^c^	—	—	—
	30-49	0.664 (0.512-0.859)	.002	—	—	—	—
**Ethnicity^d^**							
	Malay/Bumiputera	2.412 (1.526-3.813)	<.001	—	—	1.598 (0.789-3.233)	.19
	Chinese	1.459 (0.895-2.379)	.13	—	—	1.873 (0.897-3.909)	.10
	Indian	1.707 (1.002-2.909)	.049	—	—	2.887 (1.350-6.174)	.006
Urban^e^		—	—	—	—	0.776 (0.658-0.916)	.003
**Occupation^f^**							
	Student	1.40 (0.608-3.224)	.43	1.721 (0.833-3.558)	.14	0.445 (0.200-0.987)	.046
	Not employed	0.997 (0.412-2.414)	>.99	1.501 (0.692-3.259)	.30	0.409 (0.170-0.985)	.046
	Retiree	0.445 (0.173-1.147)	.094	1.404 (0.612-3.224)	.42	0.392 (0.149-1.030)	.06
	Private sector	0.649 (0.284-1.486)	.31	1.515 (0.732-3.137)	.26	0.525 (0.236-1.167)	.11
	Public servant	0.856 (0.376-1.948)	.71	1.302 (0.634-2.677)	.47	0.526 (0.239-1.157)	.11
	Self-employed	0.543 (0.23-1.276)	.16	1.725 (0.806-3.692)	.16	0.279 (0.116-0.667)	.004
**Income (RM/month)^g^**							
	<3000	1.032 (0.814-1.307)	.80	0.397 (0.320-0.493)	<.001	1.382 (1.044-1.830)	.02
	3001-6000	1.482 (1.181-1.860)	.001	0.612 (0.492-0.762)	<.001	1.449 (1.098-1.912)	.009
	6001-9000	1.405 (1.101-1.793)	.006	0.662 (0.524-0.838)	.001	1.473 (1.097-1.977)	.010
	9001-12,000	1.159 (.893-1.506)	.27	0.803 (0.620-1.040)	.10	1.168 (0.842-1.620)	.35

^a^Male respondents used as a reference.

^b^Respondents aged >50 years used as a reference.

^c^Not available.

^d^Respondents who stated their ethnicity as “other” used as a reference.

^e^Respondents from rural localities used as a reference.

^f^Respondents who stated their occupation as “other” used as a reference.

^g^Respondents whose income was ≥RM12,001/month used as a reference. A currency exchange rate of RM1=US $0.24 is applicable.

**Table 3:** Factors related to the use of COVID-19 social media information sources.

Variables		WhatsApp		Twitter		Instagram		YouTube		Telegram	
		OR (95% CI)	*P* value	OR (95% CI)	*P* value	OR (95% CI)	*P* value	OR (95% CI)	*P* value	OR (95% CI)	*P* value
Sex - Female^a^		0.829 (0.733-0.937)	.003	—^b^	—	1.477 (1.288-1.693)	<.001	0.628 (0.548-0.720)	<.001	1.492 (1.175-1.895)	.001
**Age (years)^c^**											
	18-29	0.388 (0.302-0.497)	<.001	6.264 (4.278- 9.171)	<.001	4.194 (3.204- 5.490)	<.001	0.687 (0.556- 0.850)	<.001	0.990 (0.603-1.625)	.97
	30-49	0.540 (0.429- 0.680)	<.001	2.096 (1.474- 2.982)	<.001	1.574 (1.195- 2.074)	.001	0.590 (0.477- 0.730)	<.001	1.653 (1.065- 2.566)	.03
**Ethnicity^d^**											
	Malay/Bumiputera	1.656 (1.053- 2.604)	.03	1.198 (.713-2.011)	.50	1.426 (.827-2.457)	.20	—	—	0.730 (0.329-1.621)	.44
	Chinese	0.910 (0.562-1.473)	.70	0.228 (0.128- 0.407)	<.001	0.729 (0.406-1.311)	.29	—	—	0.265 (0.102- 0.685)	.006
	Indian	1.654 (0.980-2.789)	.06	0.246 (0.126- 0.478)	<.001	1.344 (0.726-2.490)	.35	—	—	0.508 (0.188-1.374)	.18
Urban locality^e^		—	—	1.182 (1.010- 1.382)	.04	—	—	—	—	—	—
**Occupation^f^**											
	Student	0.672 (0.310-1.460)	.32	1.018 (0.455-2.278)	.97	—	—	—	—	0.516 (0.169-1.579)	.25
	Not employed	0.428 (0.190- 0.964)	.04	0.616 (0.262-1.445)	.27	—	—	—	—	0.537 (0.164-1.764)	.31
	Retiree	0.795 (0.320-1.980)	.62	0.262 (0.076- 0.897)	.03	—	—	—	—	0.110 (0.011-1.060)	.06
	Private sector	0.581 (0.269-1.256)	.17	0.726 (0.326-1.617)	.43	—	—	—	—	0.687 (0.232-2.035)	.50
	Public servant	0.783 (0.365-1.684)	.53	0.351 (0.159- 0.777)	.01	—	—	—	—	0.601 (0.205-1.758)	.35
	Self-employed	0.449 (0.202- 0.997)	.049	0.382 (0.162- 0.901)	.03	—	—	—	—	0.244 (0.070-0.854)	.03
**Income (RM/month)^g^**											
	<3000	—	—	0.537 (0.411- 0.700)	<.001	—	—	—	—	—	—
	3001-6000	—	—	0.736 (0.571- 0.948)	.02	—	—	—	—	—	—
	6001-9000	—	—	0.897 (0.684-1.175)	.43	—	—	—	—	—	—
	9001-12,000	—	—	0.875 (0.651-1.176)	.38	—	—	—	—	—	—

^a^Male respondents used as a reference.

^b^Not available.

^c^Respondents aged >50 years used as a reference.

^d^Respondents who stated their ethnicity as “other” used as a reference.

^e^Respondents from rural localities used as a reference.

^f^Respondents who stated their occupation as “other” used as a reference.

^g^Respondents whose income was ≥RM12,001/month used as a reference. A currency exchange rate of RM1=US $0.24 is applicable.

**Table 4:** Factors related to the use of COVID-19 interpersonal and health authority information sources.

Variables		MOH^a^		NSC^b^		Friends		Family	
		OR (95% CI)	*P* value	OR (95% CI)	*P* value	OR (95% CI)	*P* value	OR (95% CI)	*P* value
Sex - Female^c^		—^d^	—	1.141 (1.006- 1.296)	.04	—	—	—	—
**Age (years)^e^**									
	18-29	—	—	0.523 (0.401-0.681)	<.001	0.500 (0.408- 0.612)	<.001	0.976 (0.785-1.213)	.83
	30-49	—	—	0.950 (0.756-1.194)	.66	0.590 (0.483- 0.720)	<.001	0.652 (0.522- 0.813)	<.001
**Ethnicity^f^**									
	Malay/Bumiputera	3.521 (1.776-6.981)	<.001	1.619 (1.029-2.548)	.04	—	—	—	—
	Chinese	1.113 (0.540-2.291)	.77	0.660 (0.407-1.069)	.09	—	—	—	—
	Indian	1.465 (0.653-3.288)	.35	0.852 (0.507-1.433)	.55	—	—	—	—
Urban locality^g^		—		—		—	—	—	—
**Occupation^h^**									
	Student	—	—	1.227 (.576-2.616)	.60	—	—	—	—
	Not employed	—	—	1.001 (0.450-2.223)	>.99	—	—	—	—
	Retiree	—	—	0.826 (0.347-1.966)	.67	—	—	—	—
	Private sector	—	—	1.179 (0.556-2.501)	.67	—	—	—	—
	Public servant	—	—	1.080 (0.513-2.274)	.84	—	—	—	—
	Self-employed	—	—	0.687 (0.315-1.496)	.34	—	—	—	—
**Income (RM/month)^i^**									
	<3000	1.059 (0.716-1.567)	.77	0.783 (0.624-0.982)	.03	—	—	—	—
	3001-6000	1.630 (1.060-2.506)	.03	0.962 (0.776-1.194)	.73	—	—	—	—
	6001-9000	1.876 (1.142-3.080)	.01	1.056 (0.837-1.331)	.65	—	—	—	—
	9001-12,000	1.465 (0.871-2.462)	.15	1.034 (0.804-1.331)	.79	—	—	—	—

^a^MOH: Ministry of Health.

^b^NSC: National Security Council.

^c^Male respondents used as a reference.

^d^Not available.

^e^Respondents aged >50 years used as a reference.

^f^Respondents who stated their ethnicity as “other” used as a reference.

^g^Respondents from rural localities used as a reference.

^h^Respondents who stated their occupation as “other” used as a reference.

^i^Respondents whose income was ≥RM12,001/month used as a reference. A currency exchange rate of RM1=US $0.24 is applicable.

Table 5 focuses on the relationship between the sources of information used by respondents and their confidence in battling the COVID-19 pandemic and positive attitudes toward the Malaysian government in handling the COVID-19 crisis. The results showed that using the MOH, television, and the Malaysian NSC as information sources were significantly associated with confidence and positive attitudes among the respondents. Specifically, people who used the MOH, NSC, and television as sources of information for COVID-19 had more than 1.7 times the odds of having confidence in battling the pandemic (OR 2.8, 95% CI 1.7-4.7; *P*<.001;OR 1.9, 95% CI 1.4-2.6; *P*<.001; OR 1.8, 95% CI 1.3-2.5; *P*=.001, respectively) and positive attitudes toward the Malaysian government (OR 2.3, 95% CI 1.6-3.3; *P*<.001; OR 2.0, 95% CI 1.6-2.4; *P*<.001; OR 1.8, 95% CI 1.4-2.2; *P*<.001, respectively) compared to those who did not use these information sources. They also agreed that the Malaysian government handled the COVID-19 crisis very well. Respondents who used Instagram as their source of information were more likely to believe that the crisis was handled well by the government than those who did not (OR 1.4, 95% CI 1.1-1.8; *P*=.007), but this was not significantly associated with their confidence in battling the COVID-19 pandemic. Interestingly, people who sourced information from the World Health Organization (WHO) were less likely to harbor confidence and positive belief toward the government in handling the COVID-19 crisis (OR 0.44, 95% CI 0.3-0.6; OR 0.63, 95% CI 0.5-0.8, respectively). With regard to online news portals, WhatsApp, Twitter, and Telegram, a nonsignificant result was noted between each information source used by a respondent and confidence in battling the COVID-19 pandemic.

**Table 5:** Predicting general confidence and positive attitude toward the Malaysian government based on respondents’ sources of COVID-19–related information.

Variable (Use^a^=1)	Do you have confidence that Malaysia can win the battle against COVID-19? (Yes=1)		The government of Malaysia is handling the COVID-19 health crisis very well. (Agree=1)	
	OR (95% CI)	*P* value	OR (95% CI)	*P* value
MOH^b^	2.828 (1.714-4.667)	<.001	2.313 (1.625-3.294)	<.001
Television	1.765 (1.262-2.468)	.001	1.778 (1.430-2.210)	<.001
Online news portal	—^c^	—	—	—
NSC^d^	1.895 (1.360-2.641)	<.001	1.965 (1.600-2.415)	<.001
WhatsApp	—	—	—	—
WHO^e^	0.436 (0.312-0.609)	<.001	0.631 (0.514-0.774)	<.001
Twitter	—	—	—	—
Instagram	—	—	1.401 (1.095-1.791)	.007
Friends	0.691 (0.487-0.979)	.04	—	—
YouTube	—	—	0.687 (0.542-0.871)	.002
Family	—	—	0.734 (0.581-0.928)	.01
Radio	—	—	0.729 (0.557-0.955)	.02
Telegram	—	—	—	—

^a^Respondents who did not use the information sources were used as a reference.

^b^MOH: Ministry of Health.

^c^Not available.

^d^NSC: National Security Council.

^e^WHO: World Health Organization.

## Discussion

### Principal Findings

The findings of this study indicate that during the MCO, Malaysian people mainly relied on television for updated information related to COVID-19, followed by internet news portals. Apart from those, we also found that respondents from certain demographic groups favored other media platforms. In particular, young adults aged 18-29 years preferred referring to the WHO and Instagram for information; Malays preferred referring to the MOH, television, NSC, and WhatsApp; and young urbanites preferred referring to the WHO and Twitter. Variety in the use of information sources by demographic groups was also found in a previous study conducted in Taiwan [24].

In terms of the sources of COVID-19–related information used by our respondents, 95.1% (4614/4850) of the respondents referred to the MOH and 63.3% (3069/4850) referred to the NSC. This is because the MOH is the local authority on public health and the primary source of information on COVID-19. However, the WHO, the international governing body, was not favored by the majority of Malaysians (2295/4850, 47.3%). Possible reasons for the low preference toward using the WHO as an information source is the language barrier and a higher reliance on local health authorities among the Malaysian public.

During the MCO period, Malaysians used television as their main source of information. On television, COVID-19–related information from the MOH was consistently and constantly updated by way of daily briefings and press conferences. The public was reassured that proper actions were being taken through messages delivered by strong spokespeople, public service announcements, the use of simple language, and the use of visuals, such as informational graphics and short videos.

This study also indicated that confidence in winning the battle against the COVID-19 pandemic was more than 1.7 times higher in those who received information from the MOH, NSC, and television. Public health information has been successfully communicated using television in times of a health crisis, during which much needed knowledge for disease prevention and control can strengthen public confidence in the ability of the government to manage the pandemic [25,26].

Although the internet and social media are popular tools for information searching, this study found that the use of social media was less likely to stimulate public confidence toward government efforts in handling the COVID-19 pandemic. This likely means that social media use during the MCO was for social engagement and entertainment purposes. Our findings offer a different perspective from past studies, which found that social media, such as Facebook and Twitter, served as useful tools for raising awareness and promoting intervention measures for the H1N1 pandemic [27-32].

In conclusion, despite the abundance of media sources, traditional media was proven to be the most preferred platform for obtaining information during the COVID-19 health crisis [33]. Local authorities were also seen as champions in the dissemination of information. These findings suggest that the best way to communicate to the public in times of a health crisis is via local authorities and traditional mass media outlets.

### Limitations

This study had a few limitations, especially in the recruitment of respondents. First, the dissemination of the questionnaires was done via social media, thereby limiting the sample to those with internet connections. This may have excluded marginalized groups with limited or no internet access. Second, internet and social media users may not be a true representation of the population, as internet and social media sources are prone to bias in demographics, such as age, sex, location, and income category [34]. Compared to the Malaysian population, our study sample was somewhat skewed. According to the Department of Statistics Malaysia, the percentages of males and females in Malaysia are 51.5% and 48.5%, respectively. Our sample of 4850 respondents consisted of 2042 (42.1%) males and 2808 (57.9%) females. In terms of urban and rural distribution, 77% of Malaysians live in urban areas and 23% live in rural areas. In our study, 70% (n=3395) of respondents lived in urban areas and 30% (n=1455) lived in rural areas. Additionally, the median income for Malaysia in 2019 was RM5873 (US $1418.09) per month. In this study, 58.4% (n=2829) of respondents earned a household income of less than RM6000 (US $1448.75) per month. In terms of racial distribution in Malaysia, 69.7% of people are Malay/Bumiputera, 22.6% are Chinese, and 6.9% are Indian. In our study, 83% (n=4026) of respondents were Malay/Bumiputera, 10.5% (n=511) were Chinese, and 4.8% (n=82) were Indian [35]. In terms of employment, only 11% of Malaysians work in the public sector. However, in this study, 44.8% (n=2173) of our respondents worked in the public sector [36]. Third, the questionnaire was distributed during the first phase of the COVID-19 MCO in Malaysia. Therefore, it only illustrates the perspectives of the Malaysian public during that time. Lastly, the conclusion of this study reflects information sources and public confidence in the context of Malaysia and may not be applicable to other populations.

### Conclusions

Managing information during a pandemic is vehemently important to ensure that public health remains the top priority in the government agenda. Similarly, the public too must be equipped with accurate and timely information to keep abreast of COVID-19 news and the appropriate preventive strategies. As new scientific discoveries are made and new information becomes available, the government and relevant authorities must be able to react quickly. This is especially important as the world confronts the proliferation of misinformation, fake news, and conspiracy theories surrounding COVID-19. Old information must be replaced with new information, erroneous facts must be corrected, and public announcements and instructions must be promptly disseminated [37].

The results of this study contribute to the understanding of sources used to obtain COVID-19 information and their relationships with building public confidence in the face of a pandemic. The right tools and channels are key determinants for ensuring effective public health information delivery. The ability of health authorities to understand and utilize these tools plays an important role in building confidence among the intended public.

Additionally, the findings of this study can aid public health educators in the strategic use of information platforms and sources to effectively communicate COVID-19–related information. The results are also useful for developing strategic communication plans to cope with the increasing spread of misinformation and disinformation surrounding COVID-19 in Malaysia.

Future research is needed to understand the changing use of communication tools as the pandemic evolves. Its association and impact on public perception should also be evaluated at different stages of disease mitigation to observe any notable changes.

## Appendix

Multimedia Appendix 1 Survey Questions.

## Abbreviations

MCO: Movement Control Order
MOH: Ministry of Health
NSC: National Security Council
WHO: World Health Organization
