# Supplementary material for: Relationship Between COVID-19 Information Sources and Attitudes in Battling the Pandemic Among the Malaysian Public: Cross-Sectional Survey Study
Source: J Med Internet Res. 2020 Nov 12;22(11):e23922. doi: 10.2196/23922 (PMC7674144; doi:10.2196/23922)
Supplement: Multimedia Appendix 1 [file jmir_v22i11e23922_app1.docx]

**Questionnaire – ENGLISH**

**Citation:** Azlan AA, Hamzah MR, Tham JS, Ayub SH, Mohamad E (2020) Public knowledge, attitudes and practices towards COVID-19: A cross-sectional study in Malaysia. PLoS ONE 15(5): e0233668. https://doi.org/10.1371/journal.pone.0233668

**Demographic information**

| 1 | Please state your gender | Male  Female |
| --- | --- | --- |
| 2 | Please state your age | (open-ended question) |
| 3 | Please state your ethnicity | Malay  Bumiputera  Chinese  Indian  Others (please state) |
| 4 | Please choose the state where you are currently residing | Johor  Kedah  Kelantan  Kuala Lumpur  Melaka  Negeri Sembilan  Pahang  Perak  Perlis  Penang  Sabah  Sarawak  Selangor  Terengganu  Wilayah Persekutuan Putrajaya  Wilayah Persekutuan Labuan |
| 5 | Please state your locality | Urban  Rural |
| 6 | Please choose your occupation category | Student  Unemployed  Retiree  Private sector employee  Public/ government sector employee  Self-employed  Others (please state) |
| 7 | Please choose your HOUSEHOLD income category | RM3,000 and below per month  RM3,001 – RM6,000 per month  RM6,001 – RM9,000 per month  RM9,001 – RM12,000 per month  RM12,001 and above per month |

| 8 | What are the main MEDIA that you use to look for information on COVID-19? You may choose more than one answer. | Television  Radio  Online news portals  Whatsapp  Facebook  Twitter  Instagram  Youtube  Others (please state) |
| --- | --- | --- |
| 9 | What are the main SOURCES that you refer to when looking for information on COVID-19? You may choose more than one answer. | Ministry of Health Malaysia (MOH)  World Health Organization (WHO)  Malaysian National Security Council/ Majlis Keselamatan Negara (MKN)  Family  Friends  Others (please state) |

**Attitudes toward the COVID-19 virus (attitudes)**

| 1 | Do you have confidence that Malaysia can win the battle against the COVID-19 virus? | Yes  No |
| --- | --- | --- |
| 2 | The government of Malaysia is handling the COVID-19 health crisis very well. | Agree  Disagree  I’m not sure |
